# A Comparison of the Acute Effects of Different Forms of Yoga on Physiological and Psychological Stress: A Pilot Study

**DOI:** 10.3390/ijerph17176090

**Published:** 2020-08-21

**Authors:** Mallory Marshall, McKenzie McClanahan, Sarah McArthur Warren, Rebecca Rogers, Christopher Ballmann

**Affiliations:** Department of Kinesiology, Samford University, 800 Lakeshore Dr. Birmingham, AL 35229, USA; mmarshal@samford.edu (M.M.); mckenzie.mcclanahan29@gmail.com (M.M.); libby.mcarthur94@gmail.com (S.M.W.); rrogers1@samford.edu (R.R.)

**Keywords:** meditation, anxiety, cortisol, breathing exercises

## Abstract

Yoga is a frequently recommended stress management strategy; however, the acute stress response to varying types of yoga are not fully clear. Thus, the purpose of this study was to compare the acute effects of meditative and power yoga on indices of physiological and psychological stress. In a crossover counterbalanced design, physically active females (n = 13; age = 20.8 yrs ± 0.8, height = 164.5 cm ± 6.1, body mass = 65.0 kg ± 13.8) who did not regularly participate in yoga or mindful training enrolled in this study. Participants completed two visits each, with a standardized instructional-video 30-min yoga session with either A) meditative (Hatha style) yoga or B) power (Vinyasa style) yoga. Prior to and immediately after each yoga bout, psychological stress was assessed using the State–Trait Anxiety Inventory (STAI) questionnaire, and salivary cortisol samples were obtained to measure indices of physiological stress. State anxiety scores were significantly lower following meditative yoga (*p* = 0.047) but were not different following power yoga (*p* = 0.625). Salivary cortisol levels were significantly lower following meditative yoga (*p* = 0.020) but not following power yoga (*p* = 0.242). Results indicate that acute engagement in meditative yoga decreases markers of psychological and physiological stress, while power yoga does not impart a significant stress-relieving benefit. Findings indicate that differing types of yoga may have various stress-relieving capabilities and should be considered by individuals seeking anxiolytic benefits.

## 1. Introduction

Chronic stress is associated with numerous detrimental physiological and psychological consequences that pose a significant public health concern. High levels of stress are associated with many potentially maladaptive physiological changes that have been closely linked to an increased risk for lifestyle-related conditions and morbidities, such as coronary artery disease, type II-diabetes mellitus, and hypertension [1]. One predominant physiological, stress-related change of particular clinical significance is elevated levels of cortisol due to its control of glucose homeostasis, immune response, and regulation of substrate availability during stress [2]. Psychologically, unmanaged stress has been found to be associated with increases in a variety of mental and emotional disorders such as anxiety, depression, and low self-esteem [3]. Regular participation in physical activity has been found to reduce chronic perceived stress as well as decrease the presence of physiological stress markers (i.e., cortisol, alpha-amylase) in some individuals [4,5]. A growing body of evidence supports the use of yoga as a preferred exercise modality for reducing the detrimental effects of stress [6,7]. However, whether differing types of yoga influence acute psychological and physiological responses uniquely is not fully clear.

Different forms of yoga may have different goals, focus, mindfulness, and recruitment of muscle mass. Meditative (Hatha style) yoga focuses on mindful breathing techniques, flexibility, and meditation while power (Vinyasa style) yoga focuses on maintenance of powerful poses, muscular endurance, and has been shown to meet similar metabolic intensity criteria as moderate-intensity walking [6,8]. Both meditative and power yoga have been reported to decrease stress levels over time [9,10,11]. Smith et al. showed a 10-week hatha yoga intervention decreased anxiety and improved quality of life scores over time [9]. This is supported by reports of decreased physiologic stress outcomes (i.e., cortisol) with meditative yoga practice [12]. A 6-week program of power yoga has been shown to decrease perceived stress over time while also improving muscle strength and endurance [11]. Tay et al. showed increased heart rate variability and decreased respiration following power yoga over time with a 10-week program, which is consistent with greater relaxation and lower physiological stress [10]. While yoga programs are well supported as stress invention strategies, less is known about how various types of yoga influence acute stress responses differently.

Acutely, a single 90-min class of meditative yoga has been shown to decrease perceived stress scores in women [13]. West et al. bolstered these findings and revealed that a single bout of meditative yoga decreased perceived stress and negative affect in young women [12]. Acute engagement in power yoga has been shown to improve feelings of well-being and affect [14]. Furthermore, short and long bout durations of power yoga have been reported to decrease state anxiety levels [15]. Cortisol levels have been shown to decrease following both meditative and power yoga [16]. However, conflicting evidence has reported little to no changes in cortisol following yoga, suggesting the need for further study [17]. Overall, the effects of yoga on stress have been widely studied and the subject of numerous meta-analyses supporting its use to improve mood, decrease anxiety, lower perceived stress, and decrease physiological stress mediators [7,18]. While meditative and power yoga have been studied independently and/or in chronic regimens, little evidence exists comparing acute anxiolytic and stress relieving efficacy compared between yoga types and has been suggested as a need for study by previous investigations [19]. Furthermore, whether possible changes are manifested in psychological and/or physiological responses is unknown. Previous evidence has shown salivary cortisol and state anxiety to be sensitive to acute stress interventions [20]. Therefore, the purpose of this pilot study/brief report was to compare the acute effects of meditative and power yoga on indices of physiological (salivary cortisol) and psychological (state anxiety) stress. We hypothesized that meditative yoga would be more effective in decreasing state anxiety and salivary cortisol than power yoga.

## 2. Materials and Methods

### 2.1. Participants

Adequate sample size was determined using a priori power analysis with statistical software (G*power V 3.1.9.4). A previous investigation utilizing a 4-week Hatha yoga treatment showed improved stress levels and effect sizes of *f* = 0.32 and correlation = 0.7 [21]. Sample size was calculated using the following parameters: test = ANOVA repeated measures, *f* = 0.32, α = 0.05, β = 0.80, groups = 2, measurements = 4, correlation = 0.7. Adequate sample size needed was equated to n = 10. A convenience sample of thirteen physically active females who did not regularly practice yoga were recruited for this study from Samford University and the surrounding Birmingham, AL area. Participant suitability for exercise was determined using a physical activity readiness questionnaire (PAR-Q) [22]. If participants answered “yes” to any health questions on the PAR-Q, they were excluded before participation. Participants with factors affecting hormone levels such as hormonal disorders, adrenal gland insufficiency, pregnancy, or individuals who smoke were excluded from this study [23,24,25]. Individuals taking oral contraceptives were excluded from the cortisol analysis (n = 2). Prior to the trials, participants were asked to abstain from alcohol, caffeine, nicotine, exercise, and non-prescription medication for the 24 h before the trial [26,27,28]. Participants were also asked not to brush their teeth or use mouthwash and refrain from eating or drinking anything 1 h prior to the study to maintain saliva specimen integrity [29]. Verbal and written informed consent were obtained prior to data collection. All experimental procedures were conducted in accordance with the Declaration of Helsinki and approved by the Samford University Institutional Review Board (IRB).

### 2.2. Study Design

This study utilized a randomized crossover counterbalanced design and consisted of two pre-test post-test trials: Participants completed pre-session physiological and psychological measures, followed by a 30-min yoga session, and completed post-session measures for one power (Vinyasa style) yoga and meditative (Hatha style) yoga session. Trials for all participants took place in the same laboratory between 10:00 a.m. and 12:00 p.m. to control for circadian rhythms and fluctuation of cortisol hormone levels [30]. In addition, individual participants completed their sessions at the exact same time of day, and sessions were separated by 48 h.

### 2.3. Yoga Sessions

For the meditative yoga session, participants followed the 30-min Stress Relief portion of the DVD *Yoga for Stress Relief and Flexibility*, which is part of the *Element the Mind & Body Experience* DVD series (Element, USA). This instructional yoga session encourages and emphasizes mental focus, breathing, and relaxation. For the power yoga session, participants followed 30 min of the DVD *Power Yoga*, which is also part of the *Element the Mind & Body Experience* DVD series (Element, USA). This is a dynamic session that leads participants through a series of strong, powerful poses working large major muscle groups through standing poses and abdominal work, as well as yoga pushups and flexibility exercises. All yoga sessions were performed in isolation in a temperature and humidity stable room while wearing accustomed gym attire.

### 2.4. Psychological Measure

A psychological stress indicator was measured using the state anxiety portion of the State–Trait Anxiety Inventory (STAI). The STAI is a validated, self-reported subjective questionnaire to assess anxiety [31]. The state anxiety portion of the STAI has a total of 20 questions and indicates transient acute psychological stress to the current moment. Given the acute nature of the intervention in the current investigation, the trait anxiety portion of the STAI was omitted due to trait anxiety levels being relatively stable over time [32]. Each subset has scores that range from 20–80. Participants were instructed to read each statement and indicate how they were feeling at that exact moment by responding from 1–4, with higher numbers translating to greater anxiety. Participants completed the STAI before and after each meditative yoga and power yoga session to assess the potential psychological impact of the sessions.

### 2.5. Physiological Measures

A physiological stress indicator was measured by comparing the salivary cortisol levels before and after each yoga session. Cortisol has widely been used to measure physiological stress in a variety of settings and populations [33]. For each of the time points, a 2 mL saliva sample was collected from each participant. Pre-session samples were taken immediately before each yoga session, and post-session samples were taken immediately after each yoga session. Samples were obtained with Eagle Bioscience’s saliva collection tubes (Eagle Biosciences SKU: DCMOO63, Amherst, NH, USA), using the passive drooling technique [34]. All procedural recommendations from the manufacturer’s collection kit were followed. Vials were transported to the laboratory and placed in a freezer at −20 degrees Celsius for storage until biochemical analysis. Levels of salivary cortisol were determined using an enzyme-linked immunosorbent assay (ELISA) kit (Cayman Chemical Company, Ann Arbor, MI, USA), which had been previously validated [35]. All procedural recommendations regarding salivary analysis from the manufacture kits were followed.

### 2.6. Data Analysis

Physiological (n = 11; cortisol levels) and psychological (n = 13; STAI state anxiety scores) stress indicators in response to meditative yoga and power yoga were analyzed with Jamovi software (Version 0.9, Sydney, Australia). For all measures, a 2 × 2 [session × time] repeated-measures ANOVA was used with Tukey’s post-hoc analysis. Magnitude of main effects were calculated using eta squared (η^2^). Estimates of effect size for post-hoc comparisons were calculated using Cohen’s d effect sizes (*d*) and interpreted as: 0.2–0.49—small; 0.5–0.79—moderate; ≥0.8—large [36,37]. Significance was set at *p* ≤ 0.05 a priori. All data are presented as mean ± standard deviation (SD).

## 3. Results

**Figure 1:**
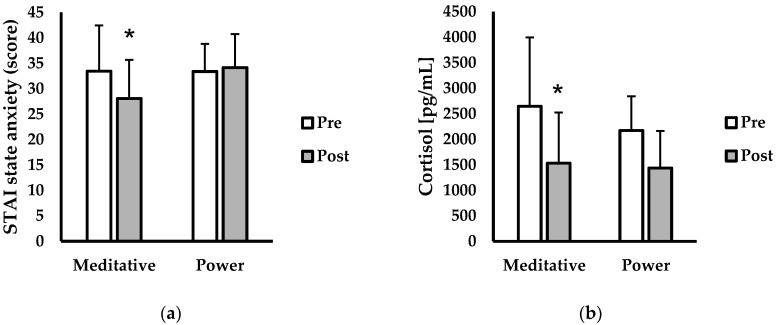
Psychological and physiological stress indices before (pre—white bars) and after (post—grey bars) either meditative or power yoga. (**a**) State–Trait Anxiety Inventory scores (STAI) (n = 13), (**b**) salivary cortisol levels (pg/mL; n = 11). Data are presented as mean ± SD. * Indicated significantly different from pre-meditative (*p* < 0.05).

Descriptive characteristics are shown in Table 1. Psychological and physiological indices of stress are presented in Figure 1. For STAI state anxiety (score; Figure 1a), there was a main effect for time (*p* = 0.015, η^2^ = 0.011), but no main effect for session (*p* = 0.993, η^2^ < 0.001), or interaction for time and session (*p* = 0.374, η^2^ = 0.001). Post-hoc analysis showed significantly lower STAI state anxiety scores post- versus pre-meditative yoga (pre-meditative = 33.4 ± 8.9, post-meditative = 27.9 ± 5.4; *p* = 0.047; *d* = 0.74). No other differences existed for STAI state anxiety scores between time points or corresponding yoga session type. For salivary cortisol (pg/mL) Figure 1b, there was a main effect for time (*p* = 0.002, η^2^ = 0.241), but no main effect for session (*p* = 0.212, η^2^ = 0.054), or interaction between time and session (*p* = 0.395, η^2^ = 0.014). Post-hoc analysis revealed that salivary cortisol levels decreased from pre- to post- meditative yoga (pre-meditative = 2645.5 pg/mL ± 1351.4, post-meditative = 1530.7 ± 670.2; *p* = 0.020; *d* = 1.10). No other differences existed for salivary cortisol levels between time points or corresponding yoga session type.

## 4. Discussion

The purpose of this pilot study was to compare the effects of an acute bout of meditative and power yoga types on psychological (state–trait anxiety) and physiological (cortisol) stress response in young, healthy women. The primary findings indicate that state anxiety and salivary cortisol significantly decrease following meditative but not power yoga. Importantly, initial levels of physiological and psychological stress were not significantly different between sessions, indicating a similar baseline level of stress at the particular moment of both yoga sessions. Thus, data suggest that there is a role for concomitant alterations of both psychological and hormonal stress responses following meditative yoga, which may have important implications when identifying potential benefits of a chronic program. While current data may be limited in generalizability to larger samples and translation to different clinical populations, these findings suggest important differences in stress-relieving capabilities of different forms of yoga, which should be considered when incorporating yoga programs into training.

Findings of decreases in state anxiety with meditative yoga are in agreement with previous literature [12,38]. While exact mechanisms for decreases in perceived anxiety were not elucidated, a large focus of meditative yoga is mindfulness and mindful breathing state [7,18]. Mindfulness, or the state of being keenly aware of the present, has been shown to increase following yoga practice and leads to decreased perceived stress [7]. Thus, the meditative yoga bout may have allowed participants to disassociate attention from external stressors after the completion of the session, improving state anxiety. Mindful breathing, another focus of meditative yoga, has also been shown to cause greater decentering from negative thoughts [39]. Interestingly, a moderate-sized decrease in state anxiety following meditative yoga in the present study was found using young, healthy individuals with relatively low-state anxiety scores. Previous investigations have suggested that individuals with high stress levels or diseased populations might be more likely to benefit from meditative yoga [40]. This suggests that meditative yoga is a potent acute stress-relieving intervention, even in mildly stressed individuals. Contrary to previous reports, an acute bout of power yoga did not reduce state anxiety [15]. This may, in part, be due to the more intense nature of the power yoga bout. While heart rate or markers of intensity were not measured in the current study, previous reports have shown heart rates between 68–71% HRmax during a 60-min power yoga session [41]. High intensity exercise has been shown to increase acute stress compared to low intensity activity [42]. Furthermore, although participants were physically active, naivety to yoga exercise may have altered the psychological stress responses. Indeed, previous investigations have reported increased state anxiety following unaccustomed activity [43]. Thus, the more intense nature of the power yoga session may have led to the abolition of any perception of stress-relieving effects. Future investigations should identify key factors, such as intensity and duration, of varying types of yoga and how they modulate stress responses.

Changes in cortisol have been repeatedly reported with meditative yoga practice, both chronically and acutely [12,44]. For example, West et al. showed that, following a single 90-min bout of meditative yoga, cortisol levels decreased, as compared to dance or a sitting control condition [12]. Important to current findings, the decreases in cortisol in participants were concomitant with decreases in state anxiety. This supports previous findings in chronic yoga programs where changes in cortisol over time successfully predicted perceived stress over a 12-week period [44]. Present findings of acute changes in cortisol may be mediated by the hallmark feature of conscious and controlled breathing during meditative yoga. Yoga breathing techniques have been shown to attenuate increases in cortisol following physical stress [45]. Additionally, similar breathing techniques have been shown to alter neural activation to stress. Mindful breathing has been reported to decrease amygdala activation, which may, in part, be responsible for attenuating the hypothalamic-pituitary-adrenal axis response to stress [46]. Further supporting this, previous investigations utilizing fMRI have shown decreased activation in the amygdala of yoga practitioners [47]. Overall, neurophysiological responses to stress with yoga are largely unclear but may play a critical role in identifying physiologic benefit of anxiolytic effects of yoga practice; more research in this area is a dire need. An acute bout of power yoga did not cause a significant change in salivary cortisol measures in participants. Previous work has shown decreases in cortisol concentrations following power yoga [48]. However, samples were obtained after a 10-min cool down period, whereas they were currently taken immediately after completing of the power yoga session. Thus, it is feasible that physiological benefits of power yoga on stress are more transient and may not be exhibited immediately following exercise. Whether different forms of yoga have different time courses for stress reliving benefits is unclear and should be the subject of subsequent investigations.

While the current pilot investigation presents novel information on stress relief following different forms of yoga, there were some limitations. Although appropriately powered, the sample in this brief report was relatively small, healthy, only female, and had low basal stress levels. In turn, this may limit the generalizability to larger populations or different groups. Given that previous evidence suggests those under greater stress may benefit from yoga practice more [40], future studies should focus on larger sample sizes with varying populations in efforts to identify specific forms of yoga, which may maximize stress relief in those individuals. Currently, markers of intensity for the activities were not measured. Thus, it is difficult to fully conclude whether differential responses to the meditative and power yoga session were due to exercise intensity or some other factor. However, stress relief with yoga practice is a multi-faceted response [7], which will need continued study to identify specific contributions of each aspect of yoga forms to positive outcomes. Lastly, there are a multitude of external factors (i.e., environmental, lighting, etc.) that could not all be controlled for and may have impacted stress responses. However, accounting for environmental stressors in real-world yoga practice is also difficult, and, thus, current results may have more generalizability.

## 5. Conclusions

In conclusion, a single meditative yoga session decreases state anxiety and cortisol levels. However, a single bout of power yoga failed to impart beneficial decreases in stress. Yoga is commonly used as a stress management strategy in a variety of populations. Current data suggest that individuals looking to maximize stress reliving benefits should consider that differing types of yoga may result in varied stress responses. Specifically, these findings suggest that meditative and mindful yoga practices meditate more favorable stress reduction while more intense and strength-focused yoga does not impart the same benefit. Future investigations focusing on different populations and varying baseline stress levels are warranted using larger sample sizes.

## Figures and Tables

**Table 1:** Descriptive characteristics (n = 13).

Characteristic	Mean ± SD
Age (y)	20.8 ± 0.8
Height (cm)	164.5 ± 6.1
Body mass (kg)	65.0 ± 13.8
BMI (kg/m^2^)	24.0 ± 3.7
